# Validation of the 18-gene classifier as a prognostic biomarker of distant metastasis in breast cancer

**DOI:** 10.1371/journal.pone.0184372

**Published:** 2017-09-08

**Authors:** Skye Hung-Chun Cheng, Tzu-Ting Huang, Yu-Hao Cheng, Tee Benita Kiat Tan, Chen-Fang Horng, Yong Alison Wang, Nicholas Shannon Brian, Li-Sun Shih, Ben-Long Yu

**Affiliations:** 1 Department of Radiation Oncology, Koo Foundation Sun Yat-Sen Cancer Center, Taipei, Taiwan; 2 Department of Research, Koo Foundation Sun Yat-Sen Cancer Center, Taipei, Taiwan; 3 Resident, Department of Medicine, Cathy General Hospital, Taipei, Taiwan; 4 National Cancer Center, Singapore (NCCS), Singapore, Singapore; 5 Department of General Surgery, Singapore General Hospital, Singapore, Singapore; 6 Duke-NUS Graduate Medical School, National University of Singapore, Singapore, Singapore; 7 Department of Medicine, Koo Foundation Sun Yat-Sen Cancer Center, Taipei, Taiwan; 8 School of Medicine, National Yang-Ming University, Taipei, Taiwan; 9 Department of Laboratory and Pathology, Koo Foundation Sun Yat-Sen Cancer Center, Taipei, Taiwan; 10 Department of Surgery, Koo Foundation Sun Yat-Sen Cancer Center, Taipei, Taiwan; University of South Alabama Mitchell Cancer Institute, UNITED STATES

## Abstract

We validated an 18-gene classifier (GC) initially developed to predict local/regional recurrence after mastectomy in estimating distant metastasis risk. The 18-gene scoring algorithm defines scores as: <21, low risk; ≥21, high risk. Six hundred eighty-three patients with primary operable breast cancer and fresh frozen tumor tissues available were included. The primary outcome was the 5-year probability of freedom from distant metastasis (DMFP). Two external datasets were used to test the predictive accuracy of 18-GC. The 5-year rates of DMFP for patients classified as low-risk (n = 146, 21.7%) and high-risk (n = 537, 78.6%) were 96.2% (95% CI, 91.1%–98.8%) and 80.9% (74.6%–81.9%), respectively (median follow-up interval, 71.8 months). The 5-year rates of DMFP of the low-risk group in stage I (n = 62, 35.6%), stage II (n = 66, 20.1%), and stage III (n = 18, 10.3%) were 100%, 94.2% (78.5%–98.5%), and 90.9% (50.8%–98.7%), respectively. Multivariate analysis revealed that 18-GC is an independent prognostic factor of distant metastasis (adjusted hazard ratio, 5.1; 95% CI, 1.8–14.1; *p* = 0.0017) for scores of ≥21. External validation showed that the 5-year rate of DMFP in the low- and high-risk patients was 94.1% (82.9%–100%) and 80.3% (70.7%–89.9%, *p* = 0.06) in a Singapore dataset, and 89.5% (81.9%–94.1%) and 73.6% (67.2%–79.0%, *p* = 0.0039) in the GEO-GSE20685 dataset, respectively. In conclusion, 18-GC is a viable prognostic biomarker for breast cancer to estimate distant metastasis risk.

## Introduction

Breast cancer is the most common cancer in women, accounting for more than one-tenth of cancers worldwide [1]. The incidence of breast cancer is rapidly rising in developing countries, and will become a major health burden in both developed and developing countries.

Owing to the advancement of breast cancer screening in recent decades, double the number of early-stage breast cancer cases are being detected as compared to the rates in the 1980s [2]. This has led to debates on the overtreatment of early-stage breast cancer [3].

Overtreatment not only increases social and family burden, but also causes unnecessary harm to patients with a favorable prognosis [4]. Early-stage breast cancer patients often receive adjuvant chemotherapy, with only a small proportion (2–20%) deriving benefit, while the others are at risk of toxic side effects [5]. Chemotherapy can lead to acute side effects (nausea and vomiting, fatigue, leukopenia, thrombocytopenia, infection, etc.) and sustained late complications (cognitive dysfunction, heart failure, or treatment-related secondary cancer) [6]. Radiotherapy increases (up to six times) the risk of death from ischemic heart disease and secondary lung cancer after 10 years [7, 8]. Therefore, cancer societies have focused on additional and new/novel adjuvant chemotherapy with fewer side effects in the past decades, and efforts to customize therapy and reduce chemotherapy for patients unlikely to benefit will probably be the main goal of future research [6].

The key to preventing early breast cancer overtreatment and providing a more individualized treatment is to utilize molecular diagnostics to determine whether the cancer is likely to be indolent or aggressive [9, 10]. In western countries, several multigene panels (Oncotype Dx, MammaPrint, and EndoPredict) have been developed to predict the possibility of distant metastasis in hormonal receptor-positive and human epidermal growth factor receptor type 2 (HER2)-negative breast cancer patients [11, 12]. These low-risk patients may omit adjuvant chemotherapy but use adjuvant hormone therapy alone [13]. Risk stratification of non-luminal breast cancer by the currently available multigene panels is limited [12]. Moreover, racial disparity of breast cancer subtypes exists [14]; whether the multigene panels developed from women of European descent are applicable to the Asian population warrants further analysis [15].

We previously reported an 18-gene classifier (18-GC) derived from a 34-gene panel initially applied to estimate the local/regional recurrence (LRR) risk in breast cancer patients after mastectomy [16]. This 18-GC includes *BLM*, *TCF3*, *PIM1*, *RCHY1*, *PTI1*, *DDX39*, *BUB1B*, *STIL*, *TPX2*, *CCNB1*, *MMP15*, *CCR1*, *NFATC2IP*, *TRPV6*, *OBSL1*, *C16ORF7*, *DTX2*, and *ENSA*. The expression of *RCHY1*, *PTI1*, *ENSA*, and *TRPV6* is associated with better tumor biology and disease control. The remaining 14 genes are associated with poor outcomes. The optimal cutoff to predict LRR *after mastectomy* was set at ≥44. Patients with scores <21 were generally free from recurrence (both locally and distantly) [17]. LRR has long been thought to be a sign of distant metastasis; patients with LRR would eventually develop systemic recurrence [18]. Thus, our gene set may not only be related to LRR, but also represent a prognostic factor for distant metastasis.

A substantial portion of breast cancer patients treated with surgery alone was deemed curable before the era of chemotherapy [19]. In this subset of patients, tumor biology is localized. We hypothesized that our LRR-gene panel can identify this disease entity, and aimed to validate the 18-GC as a prognostic factor of distant metastasis after primary breast surgery.

## Methods and patients

**Fig 1:**
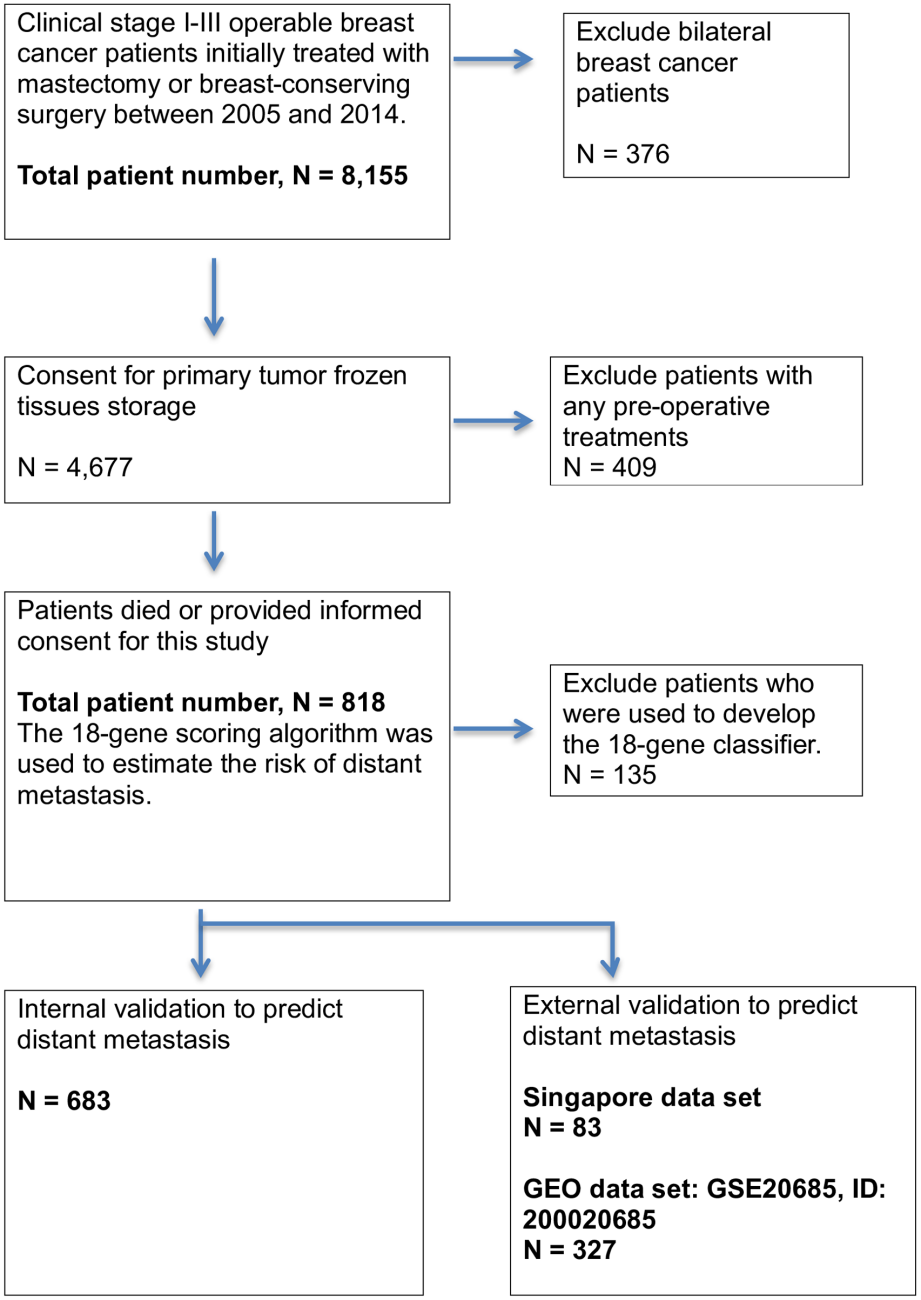
Flow of patient selection and external validation.

Between 2005 and 2014, patients with clinical stage I–III breast cancer who underwent primary surgery in a freestanding cancer center in Taiwan, and had frozen tissue of their primary tumor stored during the operation, were considered potential candidates. The flow chart for patient selection is shown in Fig 1. Events of recurrences in primary operable breast cancer were relatively small. Patients with recurrent disease were preferentially enrolled. Others were randomly selected from our database. The last follow-up date was October 30, 2016. All patients received treatment and care in accordance with contemporary, evidence-based medicine hospital practice guidelines, which are largely similar to the National Comprehensive Cancer Network guidelines. The adjuvant chemotherapy drugs used in the distant recurrent patients (n = 101) were: dose-dense ATC in 32 patients, 3 cycles of doxorubicin plus 6 cycles of cyclophosphamide/methotrexate/5-fluorouracil in 13 patients, 4 cycles of doxorubicin/ cyclophosphamide and 4 cycles of taxol or taxotere in 19 patients, 6 cycles of cyclophosphamide/doxorubicin/cyclophosphamide in 22 patients, others (mixed regimens due to intolerance of initial treatment) in 6 patients, and no adjuvant chemotherapy in 9 patients (Table 1).

**Table 1:** Baseline characteristics of the 683 patients.

Variables	Absence of metastasis (n = 582)	Presence of metastasis (n = 101)	*P*-value
Median follow-up (Months)	73.3 (0–178.5)	83.4 (25.1–198.1)	0.0301*
**Age**			**0.0449**
<40	83 (80.6%)	20 (19.4%)	
40–60	407 (87.5%)	58 (12.5%)	
>60	92 (80.0%)	23 (20.0%)	
**Surgery**			**0.019**
Mastectomy	374 (82.9%)	77 (17.1%)	
Breast conserving surgery	208 (89.7%)	24 (10.3%)	
**T stage**			**<0.0001**
T1	258 (93.5%)	18 (6.5%)	
T2	308 (81.7%)	69 (18.3%)	
T3	12 (50.0%)	12 (50.0%)	
T4	4 (66.7%)	2 (33.3%)	
**N stage**			**<0.0001**
N0	279 (93.0%)	21 (7.0%)	
N1	196 (91.6%)	18 (8.4%)	
N2	65 (74.7%)	22 (25.3%)	
N3	42 (51.2%)	40 (48.8%)	
**ER and PR status**			**0.0026**
Both Negative	187 (79.6%)	48 (20.4%)	
ER or PR (+)	395 (88.2%)	53 (11.8%)	
**HER2 overexpression**			0.0871
Negative	369 (87.0%)	55 (13.0%)	
Positive	213 (82.2%)	46 (17.8%)	
**Lymphovascular invasion**			**0.0001**
Absent/focal	450 (88.2%)	60 (11.8%)	
Prominent	132 (76.3%)	41 (23.7%)	
**Tumor grade**			**0.0135**
Grade I	64 (95.5%)	3 (4.5%)	
Grade II	194 (87.0%)	29 (13.0%)	
Grade III	324 (82.4%)	69 (17.6%)	
**Adjuvant chemotherapy**			0.5004
No	65 (87.8%)	9 (12.2%)	
Yes	517 (84.9%)	92 (15.1%)	
**Adjuvant H/T**			**0.0014**
No	203 (79.6%)	52 (20.4%)	
Yes	379 (88.6%)	49 (11.5%)	
**Adjuvant radiotherapy**			0.0239
No	119 (91.5%)	11 (8.5%)	
Yes	463 (83.7%)	90 (16.3%)	
**18-gene scores**			**<0.0001**
Low risk (<21)	142 (97.3%)	4 (2.7%)	
High risk (≥21)	440 (81.9%)	97 (18.1%)	

*The two-sample *t*-test was used to compare the mean follow-up time between the two groups.

Abbreviations: BCS, breast-conserving surgery; LVI, lymphovascular invasion; ER, estrogen receptor; PR, progesterone receptor; IHC, immunohistochemistry; R/T, radiotherapy; H/T, hormonal therapy; C/T, chemotherapy.

Eligible patients met the following inclusion criteria: (1) invasive carcinoma of the breast, (2) clinical stages I–III, (3) first treatment being surgery (modified radical mastectomy, total mastectomy and sentinel lymph node biopsy, and breast conserving surgery with sentinel node biopsy and/or axillary lymph node dissection), (4) frozen fresh tissues available, and (5) provided written informed consent. Patients who had pre-operative chemotherapy and/or radiotherapy were excluded. The study was approved by the Biobank Ethics Committee and Institutional Review Board of Koo Foundation Sun Yat-Sen Cancer Center (IRB 20131001A).

Procedures for mRNA extraction from frozen fresh tissues (tumor tissue, at least 50%) and microarray analysis have been reported previously [16, 20]. Details on the Affymetrix protocol can be found on the following website: https://www.thermofisher.com/order/catalog/product/900470?SID=srch-srp-900470. Frozen tissue samples (n = 818) from surgical specimens of primary tumors taken from patients prior to any systemic treatment were used. In brief, total RNA was isolated using Trizol (Invitrogen, Carlsbad, CA) and purified with the RNeasy Mini Kit (Qiagen, Valencia, CA). RNA quality was assessed using an RNA 6000 Nano Kit and Agilent 2100 Bioanalyzer (Agilent Technologies, Waldbronn, Germany). The RNA samples had an average RNA integrity number of 7.85 ± 0.99 (mean ± SD). Biotinylated targets were prepared according to published methods (Affymetrix, Santa Clara, CA, USA) and hybridized to the Affymetrix microarray U133 2.0 GeneChip [21]. Arrays were scanned using standard Affymetrix protocols. Raw data from Affymetrix chips were then normalized with the robust multi-array average (RMA) for background adjustment.

### Eighteen genes and scoring algorithm

18-GC development has been reported previously [17]. Each gene was assigned a weight according to the Cox proportional hazards model to assemble the 18-gene scoring algorithm (S1 and S2 Tables). The scoring algorithm is as follows:

18-gene score = 4 × TRPV6 + 3 × DDX39 + 8 × BUB1B + CCR1 + STIL + 3 × BLM + 11 × C16ORF7 + 4 × PIM1 + TPX2 + 2 × PTI1 + 2 × TCF3 + CCNB1 + DTX2 + 2 × ENSA + 5 × RCHY1 + 4 × NFATC2IP + OBSL1 + 2 × MMP15 [17]. We defined patients with scores of ≥21 as high-risk and <21 as low-risk for distant metastasis. We also evaluated the 18-gene scores as continuous variables to estimate the likelihood of distant metastasis.

### Statistical methods

Cox proportional hazards regression models were used to assess the prognostic significance of age at diagnosis, primary tumor size, the number of axillary lymph nodes involved, tumor grade, lymphovascular invasion (LVI), ER/PR status, HER2 overexpression, breast cancer subtypes, and the 18-gene score (stratified by either a categorical factor or a continuous variable). Breast cancer subtypes were classified via immunohistochemistry [22]: luminal A subtype, ER or PR (+), HER2 (-), and grade 1 to 2; luminal B subtype, ER or PR (+), HER2 (-), and grade 3. HER2 subtype means ER or PR (+/-) and HER2 (+). Triple-negative subtype represents ER (-), PR (-), and HER2 (-).

Duration of freedom from distant metastasis was defined as that from the first day of treatment to the day of diagnosis for any distant metastasis, or to the last follow-up. Probability of freedom from distant metastasis (DMFP) was calculated according to the Kaplan–Meier method. Patients with LRR or those who died before distant recurrence were censored. A log-rank test was performed to assess the statistical significance of the differences in DMFP between patient subsets. All statistical analyses (*P* < 0.05) were performed using SAS, version 9.4.

### Internal and external validation

We excluded 135 mastectomy patients who were included for the development of the 18-gene scoring algorithm in predicting LRR. Quantile normalization using the 135 patients’ gene expression levels was applied to the remaining 683 patients. These patients were then included in the internal validation for the estimation of distant metastasis risk (Fig 1).

We also used a dataset from GEO (GSE20685, ID: 200020685) from patients treated between 1991–2004, and a dataset from the National Cancer Center Singapore for external validation (Singapore CIRB 2016/2449), from which we obtained a microarray dataset without knowledge of patient outcomes. After mean shift of quantile normalization using our dataset [23], each patient was assigned a “score.” Survival analysis was performed independently by the Singapore group.

## Results

Baseline characteristics of 683 patients (232 underwent breast-conserving surgeries) in the validation group are shown in Table 1. The median follow-up interval for patients without metastasis was 73.3 (range, 0–178.5). The follow-up interval for living patients with metastasis was 83.4 (25.1–198.1) months. The age of diagnosis was between 40–60 years. The primary tumor size was usually greater than 2 cm (n = 407, 59.6%). N0 disease was the most common (n = 300, 43.9%), followed by N1 disease (n = 214, 31.3%). Most patients were ER or PR (+) (n = 448, 65.6%) and HER2 (-) (n = 424, 62.1%). Prominent lymphovascular invasion (LVI) was present in 25.3% of patients (n = 173). Tumor grade III (n = 393, 57.5%) was the most common, followed by grade II (n = 223, 32.7%).

The resection margins of all patients were negative. Adjuvant chemotherapy was administered to 89.2% (n = 609), adjuvant hormonal therapy to 62.7% (n = 428), and adjuvant radiotherapy to 81.0% (n = 553) of the patients. Treatment completion rate for adjuvant chemotherapy was 96.1% (585/609).

The patient factors strongly related to distant metastasis included age <40 or >60, more advanced tumor stage and nodal status, hormonal receptor-negative, prominent LVI, higher tumor grading, and 18-gene scores (Table 1). Treatment factors associated with distant metastasis were primary surgical type, adjuvant hormonal therapy, and adjuvant radiotherapy. Time to distant recurrence in each subtype was different (S1 Fig).

According to the 18-gene scoring algorithm, 21.4% (n = 146) of the patients were classified as low-risk (scores of <21) and 78.6% (n = 537) of the patients were classified as high-risk (scores of ≥21). The distant metastasis rates in the low- and high-risk groups were 2.7% and 18.1%, respectively (*p* < 0.0001, Table 1).

### Performance of 18-GC

**Fig 2:**
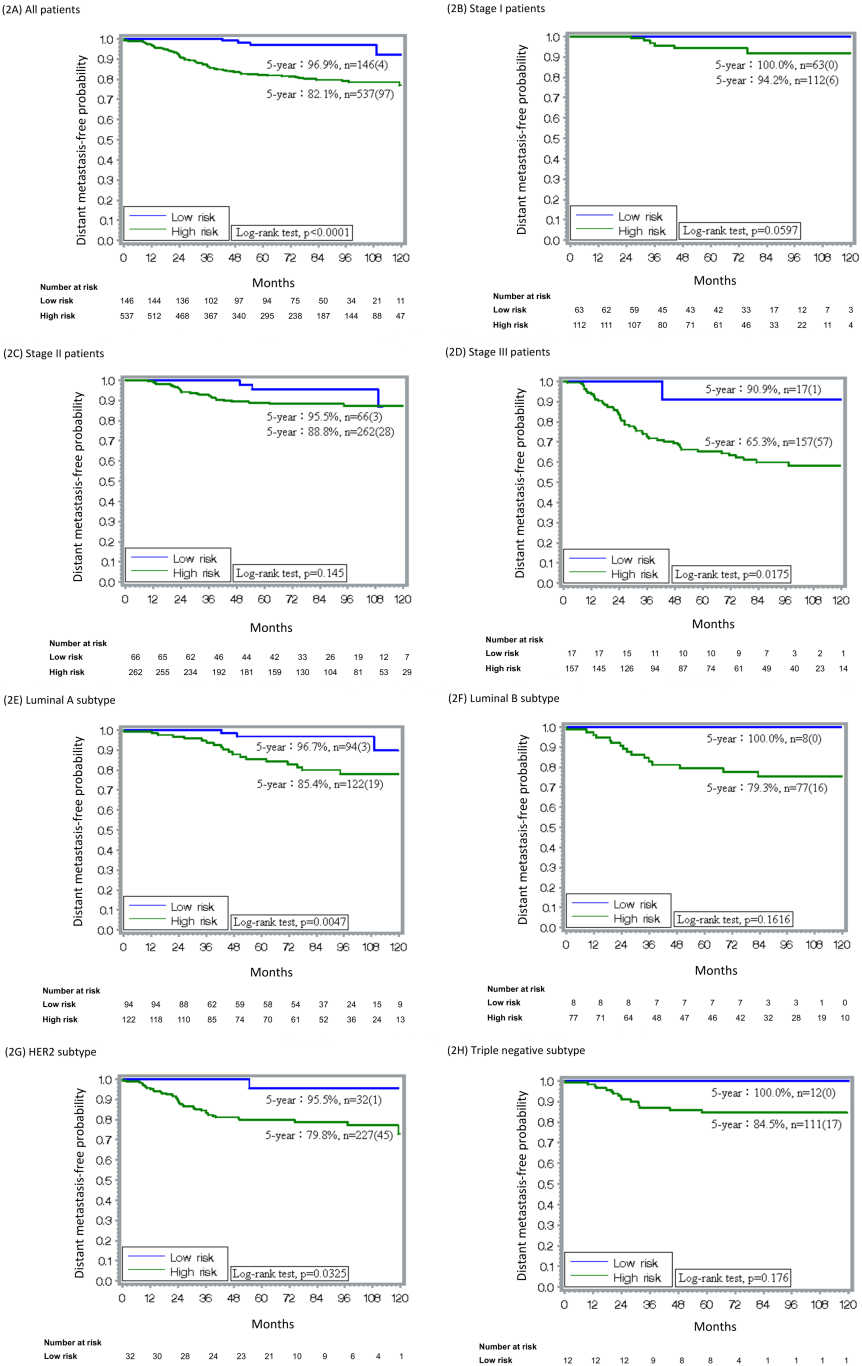
DMFP rates of low- (scores of <21) and high-risk (scores of ≥21) patients by 18-GC. (A) All patients (n = 683), (B) stage I (n = 175), (C) stage II (n = 328), (D) stage III (n = 174), (E) luminal A subtype (n = 216), (F) luminal B subtype (n = 85), (G) HER2 subtype (n = 259), and (H) triple-negative subtype (n = 123).

The 5-year rates of DMFP in the low-risk and high-risk groups were 96.9% (95% CI, 90.8%–99.0%) and 82.1% (78.3%–85.3%, *p* < 0.0001), respectively (Fig 2A). The percentages of the low-risk group analyzed with 18-GC in stages I, II, and III were 36.0% (63/175), 20.1% (66/328), and 9.8% (17/174), respectively (Fig 2B–2D). In the low-risk group (scores of <21), the 5-year rates of DMFP in stages I, II, and III were 100%, 95.5% (83.0%–98.8%), and 90.9% (50.8%–98.7%), respectively. In the high-risk group (scores of ≥21), the 5-year rates of DMFP in stages I, II, and III were 94.2% (86.7%–97.6%), 88.8% (84.0%–92.3%), and 65.3% (56.8%–72.5%), respectively.

Regarding breast cancer subtypes by immunohistochemistry **(**Fig 2E–2H), 31.6% (n = 216) of patients were a luminal-A subtype, 12.4% (n = 85) were luminal-B subtype, 37.9% (n = 259) were HER2 subtype, and 18.0% (n = 123) were triple-negative subtype.

The proportion of low-risk patients in luminal-A was 43.5% (94/216), luminal-B was 9.4% (8/85), HER2-enriched was 12.4% (32/259), and triple-negative subtype was 9.8% (12/123; Fig 2E–2H). The rate of 5-year DMFP of the low-risk group, including those of stage I–III patients, was excellent: luminal-A subtype, 96.7% (87.4%–99.2%); luminal-B subtype, 100%; HER2-enriched subtype, 95.5% (71.9%–99.3%), and triple-negative subtype, 100%. Among the high-risk group, the 5-year DMFP rate in the luminal-A subtype was 85.4% (76.4%–91.1%), luminal-B was 79.3% (67.4%–87.3%), HER2-enriched subtype was 79.8% (73.6%–84.7%), and triple-negative subtype was 84.5% (75.9%–90.3%).

### Univariate and multivariate analysis

Univariate analysis for distant metastasis by Cox proportional hazards regression revealed that age, subtype, tumor stage, nodal stage, tumor grade, LVI, and adjuvant treatments were prognostic factors. The hazard ratio (HR) was 7.1 (95% CI, 2.6–19.2) for patients with 18-gene scores of ≥21 compared to patients with scores of <21 (HR 1.0). In the multivariate analysis, the hazard ratio was 4.9 (95% CI, 1.8–13.5; Table 2). Other risk factors independently related to distant metastasis included T stage (T2, HR 1.8, [1.0–3.1]; T3-T4, HR 3.2 [1.5–6.8]) and N stage (N2, HR 3.4 [1.8–6.6]; N3, HR 6.0, [3.3–10.9]). No adjuvant chemotherapy increased distant metastasis risk with a hazard ratio of 2.6 (95% CI, 1.2–5.7; Table 2). Breast cancer subtypes by immunohistochemistry were prognosticative after univariate analysis, but not significant after multivariate analysis.

**Table 2:** Univariate and multivariate analyses of factors associated with distant metastasis based on Cox proportional hazards models (n = 683).

Variable		Hazard ratio (95% CI) (Crude)	*P*-value	Hazard ratio (95% CI) (Adjusted)*	*P*-value
**Age**	<40	0.9 (0.5, 1.6)	0.6424	0.7 (0.4, 1.3)	0.3121
	40–60	**0.5 (0.3, 0.9)**	**0.0153**	**0.5 (0.3, 0.9)**	**0.0138**
	>60	1		1	
**IHC Subtype**	TNBC	1.3 (0.7, 2.5)	0.3654		
	HER2	**1.9 (1.1, 3.2)**	**0.0132**		
	Luminal B	**1.9 (1.0, 3.7)**	**0.0464**		
	Luminal A	1			
**T stage**	T2 vs. T1	**2.8 (1.6, 4.6)**	**0.0001**	**1.8 (1.0, 3.1)**	**0.0425**
	T3-4 vs. T1	**8.0 (4.0, 16.1)**	**<0.0001**	**3.2 (1.5, 6.8)**	**0.0018**
**N stage**	N1 vs. N0	1.2 (0.6, 2.2)	0.6537	1.1 (0.6, 2.2)	0.7591
	N2 vs. N0	**3.8 (2.1, 7.0)**	**<0.0001**	**3.4 (1.8, 6.6)**	**0.0003**
	N3 vs. N0	**8.4 (5.0, 14.3)**	**<0.0001**	**6.0 (3.3, 10.9)**	**<.0001**
**Tumor grade**	III vs. I-II	**1.7 (1.1, 2.6)**	**0.0109**		
**LVI**	No/focal	1	**0.0002**		
	Prominent	**2.1 (1.4, 3.2)**			
**Adjuvant H/T**	No vs. Yes	**1.9 (1.3, 2.8)**	**0.0011**		
**Adjuvant R/T**	No vs. Yes	**0.5 (0.3, 1.0)**	**0.047**		
**Adjuvant C/T**	No vs. Yes	0.9 (0.5, 1.8)	0.8383	**2.6 (1.2, 5.7)**	**0.0147**
**18-gene score**	≥21 vs. <21	**7.1 (2.6, 19.2)**	**0.0001**	**4.9 (1.8, 13.5)**	**0.0023**

*Note: Using the stepwise method for model selection, breast cancer subtypes were not significantly associated with distant recurrence in the final model of multivariate analyses.

Abbreviation: refer to Table 1.

**Fig 3:**
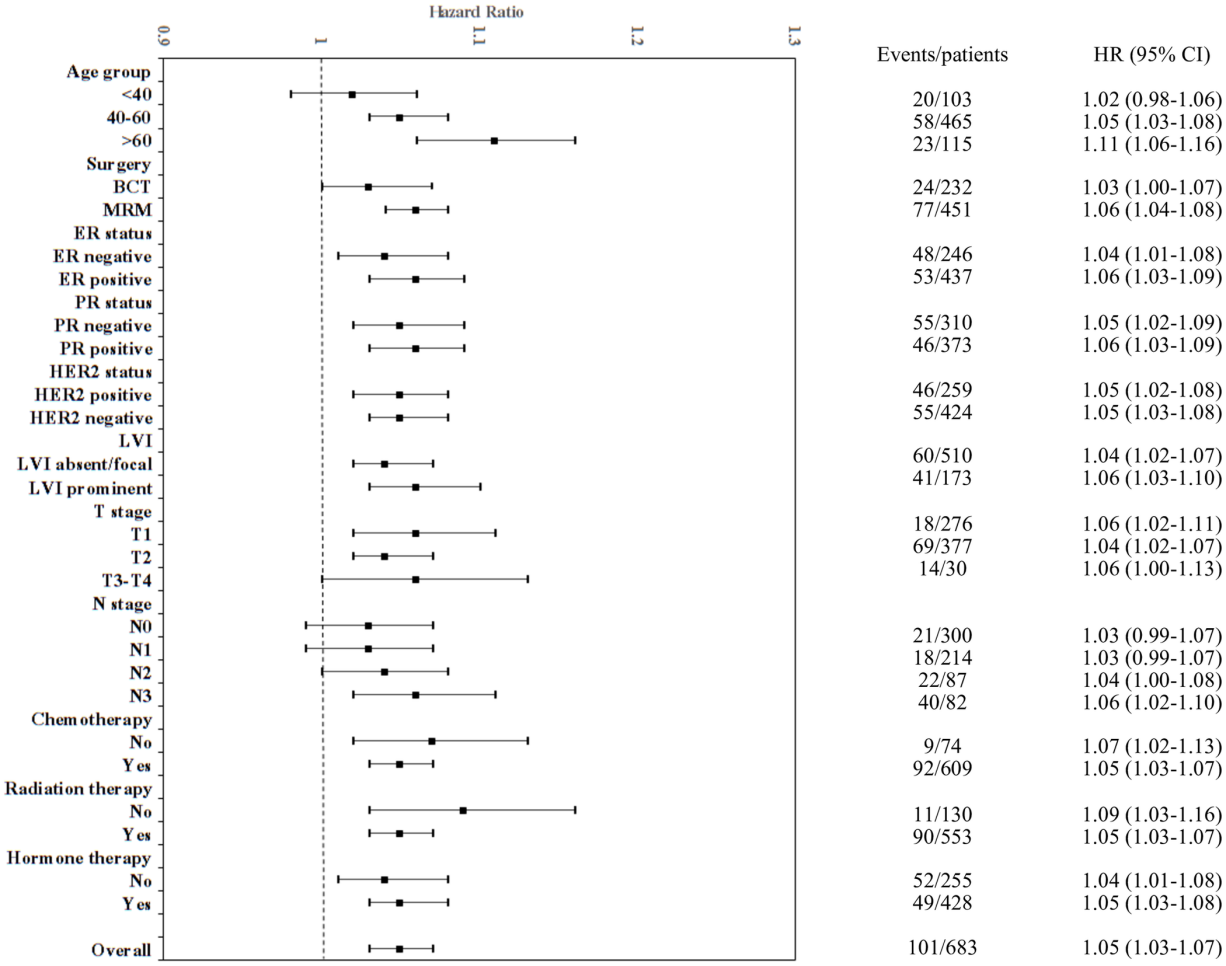
Hazard ratio forest plots for each subgroup. 18-GC as a continuous variable in the prediction of distant recurrence.

Using the 18-gene score as a continuous variable, 18-GC was highly associated with distant recurrence in the exploratory subgroup analyses **(**Fig 3). This observation was true across different age groups (40–60 and >60); T1, T2, and T3-T4; subtypes (ER, PR, and HER2 status); and treatment modalities (chemotherapy, radiotherapy, and hormone therapy). The hazard ratio for distant recurrence risk was 1.05 (1.03–1.07) for a score increment of 1. If we combine the training data set (n = 135) and the validation data set (n = 683) together, the hazard ratio for distant recurrence risk was 1.08 (1.06–1.10) (S2 Fig).

### External validation

**Fig 4:**
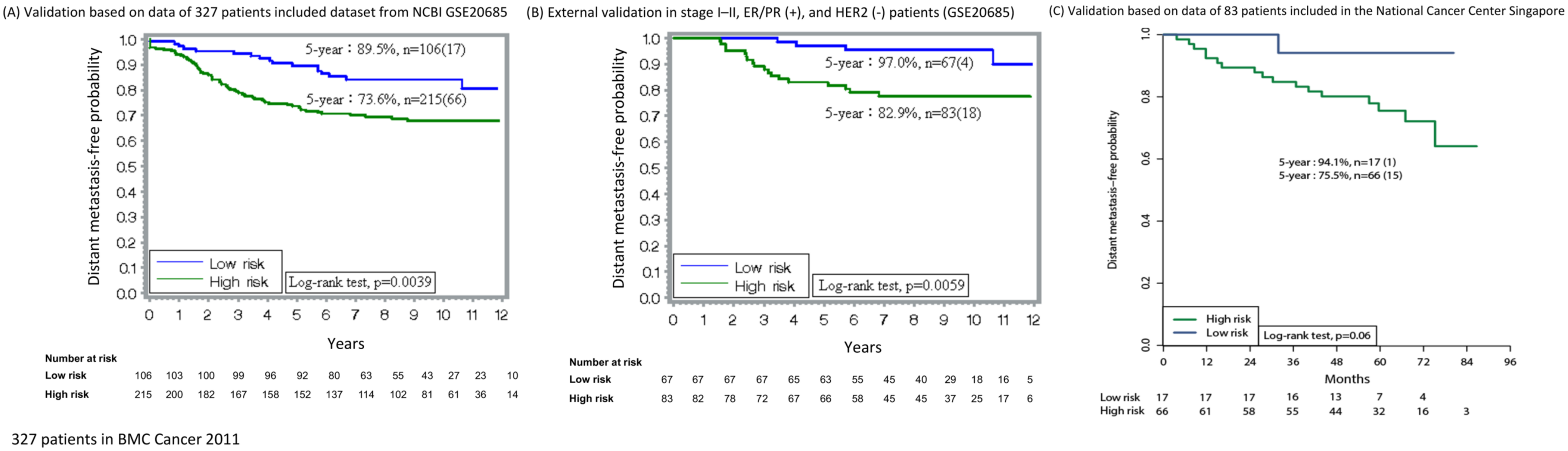
External validation of 18-GC. (A) Validation based on data of 327 patients included dataset from NCBI GSE20685, ID: 200020685. Time to distant metastasis by the 18-gene scores: The 5-year rate of DMFP in patients with scores of <21 was 89.5% (95% CI, 81.9%–94.1%) and the rate in patients with scores of ≥21 was 73.6% (67.2%–79.0%). (B) External validation in stage I–II, ER/PR (+), and HER2 (-) patients (GSE20685). Time to distant metastasis by 18-gene scores (n = 150): the 5-year DMFP (95% CI) <21: 97.0% (88.5%–99.2%), n = 67 (4); ≥21: 82.9% (72.9%–89.5%), n = 83 (18). (C) Validation based on data of 83 patients included in the National Cancer Center Singapore. Five-year rates of DMFP in the low- and high-risk patients are shown.

Using the GEO (GSE20685) dataset for external validation, stage distribution was found to be 21.1% (n = 69), 45.0% (n = 147), 31.5% (n = 103), and 2.4% (n = 8) in stages I, II, III, and IV, respectively. In this study, 62.4% (204) of the patients were ER (+) and 22.9% (75) of the patients overexpressed HER2. Baseline characteristics are shown in S3 Table. Fig 4A shows the distant recurrence probability according to the 18-gene scores. Recurrent patients usually had higher 18-gene scores. The 5- and 10-year rates of DMFP in patients with scores of <21 (n = 106) were 89.5% (81.9%–94.1%) and 84.1% (75.3%–90.0%), respectively. The 5- and 10-year rates in patients with scores of ≥21 (n = 215) were 73.6% (67.2%–79.0%) and 67.9% (61.0%–73.9%), respectively. Fig 4B shows the results of luminal breast cancer with stage I–II diseases stratified according to 18-GC. The 5- and 10-year rates of DMFP in the low-risk patients (n = 67) were 97.0% (88.5%–99.2%) and 95.3% (86.2%–98.5%), respectively. The 5- and 10-year rates in the high-risk (n = 83) patients were 82.9% (72.9%–89.5%) and 77.4% (66.5%–85.2%), respectively.

The Singapore dataset included 55 patients of ER/PR (+), 14 patients of HER2 (+) and 14 patients of triple negative; 71/83 had mastectomies. The 5-year rates of DMFP in patients with scores of <21 and ≥21 were 94.1% (82.9–100%) and 80.3% (70.7–89.9%), respectively. Low-risk patients were mainly of a luminal subtype (Fig 4C). All HER2 (+) and triple-negative patients were classified into the high-risk group.

## Discussion

18-GC is a multifunctional gene panel that is associated with cell cycle and proliferation (*DDX39*, *BUB1B*, *STIL*, *TPX2*, *CCNB1*), oncogenic process (*BLM*, *TCF3*, *PIM1*, *RCHY1*, *PTI1*), inflammation and immune response (*CCR1*, *NFATC2IP*), cell-cell interaction (*TRPV6*, *OBSL1*, *MMP15*), apoptosis (*C16ORF7*, *DTX2*), and metabolism (*ENSA*) [16]. Our 18-GC prognosticates distant metastasis regardless of cancer subtype, stage, age, and treatment modalities. The 5-year rates of DMFP were 96.9% and 82.1% in the low- and high-risk groups, respectively (Fig 2A). That 18-GC can partition the low- and high-risk groups of distant metastasis was corroborated by external validation datasets (Fig 4A–4C).

Adjusted by other clinical and pathological variables, 18-GC is an independent prognostic factor for distant metastasis with a hazard ratio of 4.9 (95% CI, 1.8–13.5) for patients with scores of ≥21 versus <21 (Table 2). Forest plot analyses confirmed that 18-GC was significantly related to distant metastasis in nearly all subgroups. Using the 18-gene score as a continuous variable, the hazard ratio for distant metastasis was 1.05 (95% CI, 1.03–1.07) per score increment. 18-GC can be a prognostic biomarker for distant metastasis regardless of adjuvant hormonal therapy, chemotherapy, and radiotherapy (Fig 3).

The study group (135 training, 683 validation) was selected from an initial cohort of 8,155 patients, which represented a randomly selected breast cancer population in a free-standing cancer center where about one-tenth of breast cancer patients in Taiwan are treated [24, 25]. Unlike other multigene panel trials that focused on a specific subtype or stage of breast cancer, our study shows that 18-GC is a sound classifier predicting favorable or unfavorable prognosis regarding distant metastasis in a general breast cancer patient population.

The distant metastatic rate was higher in our study group than in our initial group because patients with recurrences were preferentially enrolled (Fig 1). However, the result derived from this cohort is consistent with the common consensus that 1) higher T and N stages increase the likelihood of distant metastasis; 2) adjuvant treatments decrease the likelihood of distant metastasis; 3) triple-negative, HER2, and luminal-B subtypes are more aggressive than luminal-A breast cancer with higher 18-gene scores and recurrence rates (Table 2).

Overall, the 18-GC can identify 21% of low-risk breast cancer patients with a 5-year distant metastasis rate of <4%, regardless of stages and subtypes. There are three unique characteristics of the 18-GC: 1) it is the first Asian-based gene expression profiling validated in a large and diverse breast cancer population; 2) it could stratify the risk of relapse in both luminal and non-luminal breast cancer, although the proportion of low-risk patients with non-luminal cancer is small, and 3) it can simultaneously estimate the likelihood of LRR and distant metastasis.

During the past 15 years, there has been a consensus that early-stage breast cancer is a heterogeneous disease with different molecular subtypes and prognoses [9, 26, 27]. Several multigene panels for early-stage breast cancer have been developed [12]. The subsequent multigene panels (Prosigna, EndoPredict, Breast Cancer Index) were claimed to be better than the former ones (Oncotype Dx, MammaPrint, Genomic Grade Index) for the prediction of late distant metastasis [28, 29]. These new panels focus mainly on luminal-like and node-negative breast cancer and could lead to the possibility of omitting chemotherapy in the low-risk group.

However, the possibility of stratifying the risk of relapse in non-luminal breast cancer patients remained an unsolved question. For example, the prognostic risk discrimination of the 70-gene and 76-gene panels is efficient among ER (+) patients, but <5% of ER (-) patients are classified as being in the low-risk group [9, 10]. A relatively high false-negative rate (>50%) for the HER-2 subtype by the 21-gene panel was also noted [30]. The first-generation genomic panels rely largely on quantification of proliferation-related genes to determine the prognosis of ER (+) disease [31]. Consequently, these panels are limited by their clinical utility in other breast cancer subtypes. The gene expression profiles related to immune response and stromal invasion have prognostic value for ER (-) and high proliferative ER (+) breast cancer patients [32–35]. However, those classified by second-generation genomic panels as the low-risk group had a 5-year relapse rate of up to 20%.

In this study, 18-GC identified 12% HER2 (+) and 10% triple-negative breast cancer patients as the low-risk group with a 5-year distant metastasis rate of 5% and 0%, respectively. This result opens the possibility of stratifying the relapse risk of non-luminal breast cancer patients. If this can be achieved, reduction of the chemotherapy dosage in this subset may be possible. Additionally, our study revealed that patients with high 18-gene scores would have a higher risk of distant metastasis than those with low scores; the 5-year rate of DMFP was 20%–30% regardless of treatments, and therefore, dose-dense chemotherapy or a novel clinical trial may be considered in these patients.

Although our research indicates great improvement in classifying breast cancer, there are limitations to our study. First, it is a single-centered, retrospective study. Up to 89% of the patients underwent adjuvant chemotherapy, and 81% underwent radiotherapy. Although our data showed that 18-GC is a prognostic tool in different treatment modalities, it is still hard to surmise that adjuvant therapy can be omitted in the low-risk group. However, our data would enable the identification of a group of high-risk patients who may benefit from dose-dense chemotherapy, higher dose of radiotherapy, or novel clinical trials. The 5-year distant metastasis rate in the low-risk group was only 2.7% (4/146). Meta-analyses of long-term outcomes have suggested that adjuvant chemotherapy may reduce the recurrence risk by 30%–50% [36]. Even if we assumed the greatest odds, say 50%, the initial distant metastasis rate without chemotherapy in the low-risk group would be around 5%. Thus, only 3% may benefit from the procedure, and it could expose the other 95% of patients to the toxic effect of chemotherapy in the low-risk group. A prospective, multi-centered study is warranted to validate whether the low-risk-group patients can forego chemotherapy for luminal breast cancer and whether the chemotherapy dosage may be decreased in non-luminal breast cancer. Second, some selection bias existed in our study. For the mRNA microarray, we preferentially enrolled those patients with recurrence or mortality. Therefore, our distant metastasis rate may overestimate the recurrence risk.

In conclusion, the risk stratification limitations of clinical and pathological parameters still leave a significant number of patients with breast cancer at risk of overtreatment or suboptimal treatment. However, the 18-GC can predict LRR and distant metastasis simultaneously, and will aid the advancement into a new era of precision medicine and breast cancer treatment.

## Supporting information

S1 Table Eighty-four probesets in 30 genes of interest. (DOCX)

S2 Table Hazard ratio composition of the 18-gene scoring algorithm: Univariate analysis versus multivariate analysis. A hazard ratio of <1 was counted as one point in multivariate analysis.
(DOCX)

S3 Table Baseline characteristics of patients in the GEO (GSE20685) dataset. (DOCX)

S1 Fig Time to distant recurrence in each subtype; boxplots from 25th to 75th percentiles. (TIF)

S2 Fig Hazard ratio forest plot for each subgroup to predict distant recurrence by 18-gene score (as continuous variable) in all patients (n = 818). (TIF)
