# Supplementary material for: Validation of the 18-gene classifier as a prognostic biomarker of distant metastasis in breast cancer
Source: PLoS One. 2017 Sep 8;12(9):e0184372. doi: 10.1371/journal.pone.0184372 (PMC5590926; doi:10.1371/journal.pone.0184372)
Supplement: S1 Table — (DOCX) [file pone.0184372.s001.docx]

**S1 Table. Eighty-four probesets in 30 genes of interest:** Our 18-gene classifier was derived from 34 genes of interest (GOI) in 2006 using U95 arrays. Only 30 GOI could be identified in U133 plus 2.0 arrays. The probsets of 30 GOI between U95 and U133 arrays were listed.

|  | **Probeset_U133** | **GENE_U95** |  | **Probeset_U133** | **GENE_U95** |
| --- | --- | --- | --- | --- | --- |
| 1 | 1560942_at | PGD | 43 | 229604_at | CMAH |
| 2 | 1560943_s_at | PGD | 44 | 1554862_at | CMAH |
| 3 | 201118_at | PGD | 45 | 212832_s_at | CKAP5 |
| 4 | 202140_s_at | CLK3 | 46 | 1555278_a_at | CKAP5 |
| 5 | 238072_at | CLK3 | 47 | 201584_s_at | DDX39 |
| 6 | 202648_at | TCF3 | 48 | 215732_s_at | DTX2 |
| 7 | 209151_x_at | TCF3 | 49 | 179_at | DTX2 |
| 8 | 209152_s_at | TCF3 | 50 | 210707_x_at | DTX2 |
| 9 | 209153_s_at | TCF3 | 51 | 225527_at | CEBPG |
| 10 | 210776_x_at | TCF3 | 52 | 204203_at | CEBPG |
| 11 | 213730_x_at | TCF3 | 53 | 202760_s_at | AKAP2 |
| 12 | 213731_s_at | TCF3 | 54 | 202759_s_at | AKAP2 |
| 13 | 213732_at | TCF3 | 55 | 226694_at | AKAP2 |
| 14 | 213809_x_at | TCF3 | 56 | 1553502_a_at | AKAP2 |
| 15 | 213811_x_at | TCF3 | 57 | 205098_at | CCR1 |
| 16 | 215260_s_at | TCF3 | 58 | 205099_s_at | CCR1 |
| 17 | 216647_at | TCF3 | 59 | 212809_at | NFATC2IP |
| 18 | 228052_x_at | TCF3 | 60 | 212808_at | NFATC2IP |
| 19 | 214471_x_at | LHB | 61 | 229235_at | NFATC2IP |
| 20 | 205781_at | C16ORF7 | 62 | 238130_at | NFATC2IP |
| 21 | 205733_at | BLM | 63 | 217526_at | NFATC2IP |
| 22 | 212743_at | RCHY1 | 64 | 217527_s_at | NFATC2IP |
| 23 | 212749_s_at | RCHY1 | 65 | 210105_s_at | FYN |
| 24 | 214281_s_at | RCHY1 | 66 | 212486_s_at | FYN |
| 25 | 235300_x_at | RCHY1 | 67 | 216033_s_at | FYN |
| 26 | 213477_x_at | PTI1 | 68 | 203755_at | BUB1B |
| 27 | 217528_at | CLCA2 | 69 | 202659_at | PSMB10 |
| 28 | 206164_at | CLCA2 | 70 | 221486_at | ENSA |
| 29 | 206165_s_at | CLCA2 | 71 | 221487_s_at | ENSA |
| 30 | 206166_s_at | CLCA2 | 72 | 228851_s_at | ENSA |
| 31 | 212775_at | OBSL1 | 73 | 228852_at | ENSA |
| 32 | 212776_s_at | OBSL1 | 74 | 202596_at | ENSA |
| 33 | 213946_s_at | OBSL1 | 75 | 208743_s_at | YWHAB |
| 34 | 214928_at | OBSL1 | 76 | 217717_s_at | YWHAB |
| 35 | 227573_s_at | OBSL1 | 77 | 217718_s_at | YWHAB |
| 36 | 227574_at | OBSL1 | 78 | 205339_at | STIL |
| 37 | 238776_x_at | OBSL1 | 79 | 209350_s_at | GPS2 |
| 38 | 210052_s_at | TPX2 | 80 | 243883_at | MMP15 |
| 39 | 1559405_a_at | TRPV6 | 81 | 203365_s_at | MMP15 |
| 40 | 206827_s_at | TRPV6 | 82 | 214710_s_at | CCNB1 |
| 41 | 205518_s_at | CMAH | 83 | 228729_at | PIM1 |
| 42 | 210571_s_at | CMAH | 84 | 209193_at | PIM1 |
