# Supplementary material for: Validation of the 18-gene classifier as a prognostic biomarker of distant metastasis in breast cancer
Source: PLoS One. 2017 Sep 8;12(9):e0184372. doi: 10.1371/journal.pone.0184372 (PMC5590926; doi:10.1371/journal.pone.0184372)
Supplement: S2 Table — A hazard ratio of <1 was counted as one point in multivariate analysis. (DOCX) [file pone.0184372.s002.docx]

**S2 Table. Hazard ratio composition of the 18-gene scoring algorithm: univariate analysis versus multivariate analysis. A hazard ratio of <1 was counted as one point in multivariate analysis.** Any probeset correlated with LRR in our development study were retained in the analysis. Top 18 GOI having the best sensitive and specificity to identify LRR patients were retained to form the predefined scoring algorithm.

| **Parameter** | **Single_Hazards Ratio** | **Pr > ChiSq** | **Multiple_Hazards Ratio** | **Pr > ChiSq** |
| --- | --- | --- | --- | --- |
| TRPV6 | 4.024 | 0.0059 | 3.925 | 0.0193 |
| DDX39 | 6.611 | 0.0006 | 3.401 | 0.1006 |
| BUB1B | 10.399 | 0.0016 | 7.691 | 0.1396 |
| CCR1 | 4.246 | 0.0043 | 0.352 | 0.2403 |
| STIL | 4.415 | 0.0165 | 0.682 | 0.6585 |
| BLM | 7.231 | 0.0014 | 3.12 | 0.1485 |
| C16ORF7 | 5.9 | 0.0042 | 11.231 | 0.0049 |
| PIM1 | 3.727 | 0.0755 | 3.904 | 0.1709 |
| TPX2 | 9.353 | 0.0025 | 0.683 | 0.8181 |
| PTI1 | 3.387 | 0.0104 | 2.087 | 0.2978 |
| TCF3 | 2.698 | 0.1097 | 2.186 | 0.2593 |
| CCNB1 | 6.861 | 0.0093 | 0.757 | 0.8682 |
| DTX2 | 2.85 | 0.0908 | 0.417 | 0.2222 |
| ENSA | 2.17 | 0.0659 | 2.224 | 0.1854 |
| RCHY1 | 4.756 | 0.002 | 5.038 | 0.0092 |
| NFATC2IP | 4.71 | 0.0123 | 4.188 | 0.0699 |
| OBSL1 | 1.703 | 0.2404 | 0.506 | 0.2284 |
| MMP15 | 6.209 | 0.0032 | 1.835 | 0.4305 |
