# Supplementary material for: Validation of the 18-gene classifier as a prognostic biomarker of distant metastasis in breast cancer
Source: PLoS One. 2017 Sep 8;12(9):e0184372. doi: 10.1371/journal.pone.0184372 (PMC5590926; doi:10.1371/journal.pone.0184372)
Supplement: S3 Table — (DOCX) [file pone.0184372.s003.docx]

**S3 Table. Baseline characteristics of patients in the GEO (GSE20685) dataset.**

| Variables |  | Patient # | % |
| --- | --- | --- | --- |
| Age | < 50 yr. | 203 | 62.1 |
|  | > 50 yr. | 124 | 37.9 |
| TNM stage | I | 69 | 21.1 |
|  | II | 147 | 45.0 |
|  | III | 103 | 31.5 |
|  | IV | 8 | 2.4 |
| ER status | Positive | 204 | 62.4 |
|  | Negative | 123 | 37.6 |
| HER amplified | Positive | 75 | 22.9 |
|  | Negative | 252 | 77.1 |
