# Supplementary figures and images for: Validation of the 18-gene classifier as a prognostic biomarker of distant metastasis in breast cancer
Source: PLoS One. 2017 Sep 8;12(9):e0184372. doi: 10.1371/journal.pone.0184372 (PMC5590926; doi:10.1371/journal.pone.0184372)

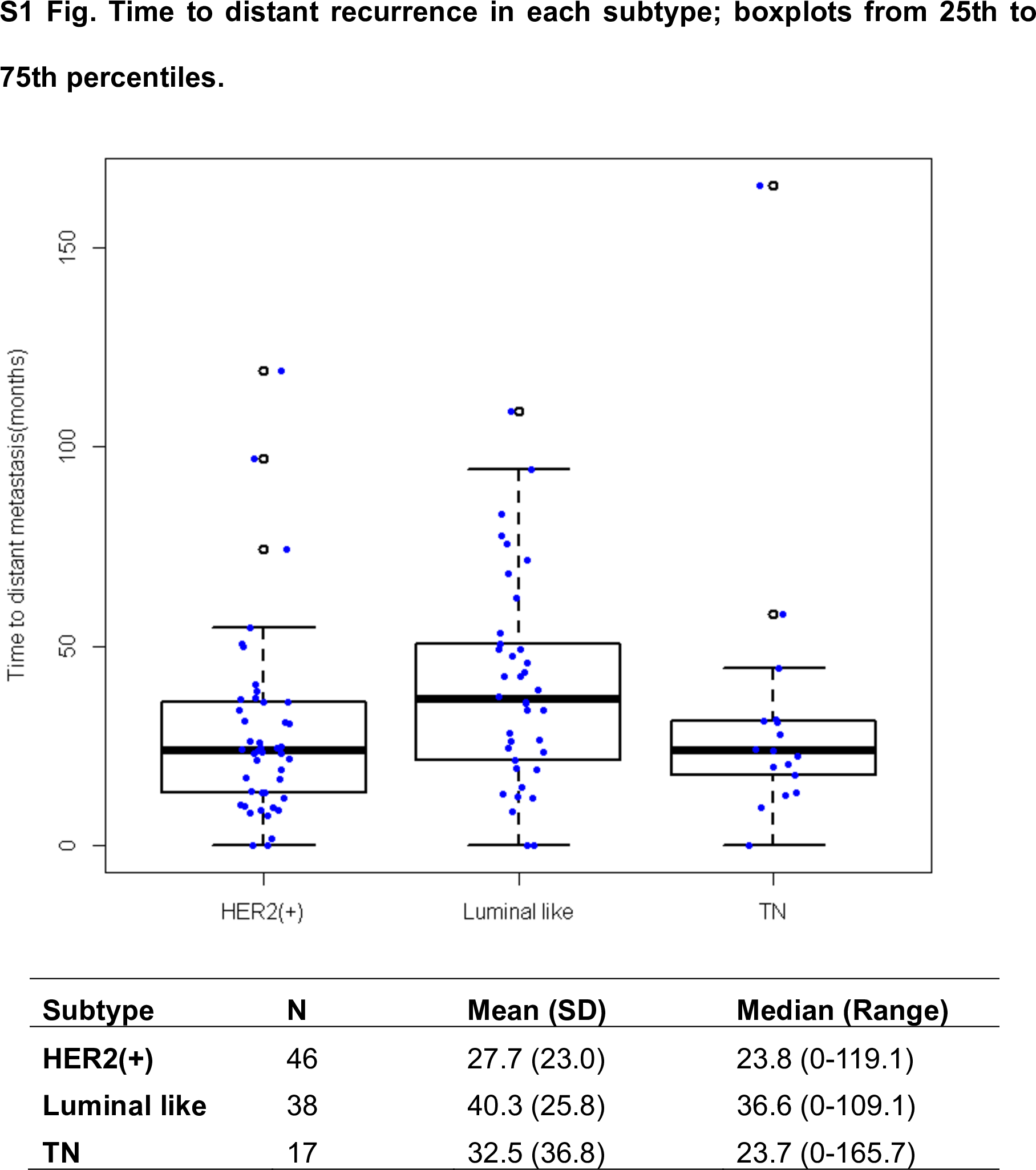

Supplement: S1 Fig — (TIF) [file pone.0184372.s004.tif]

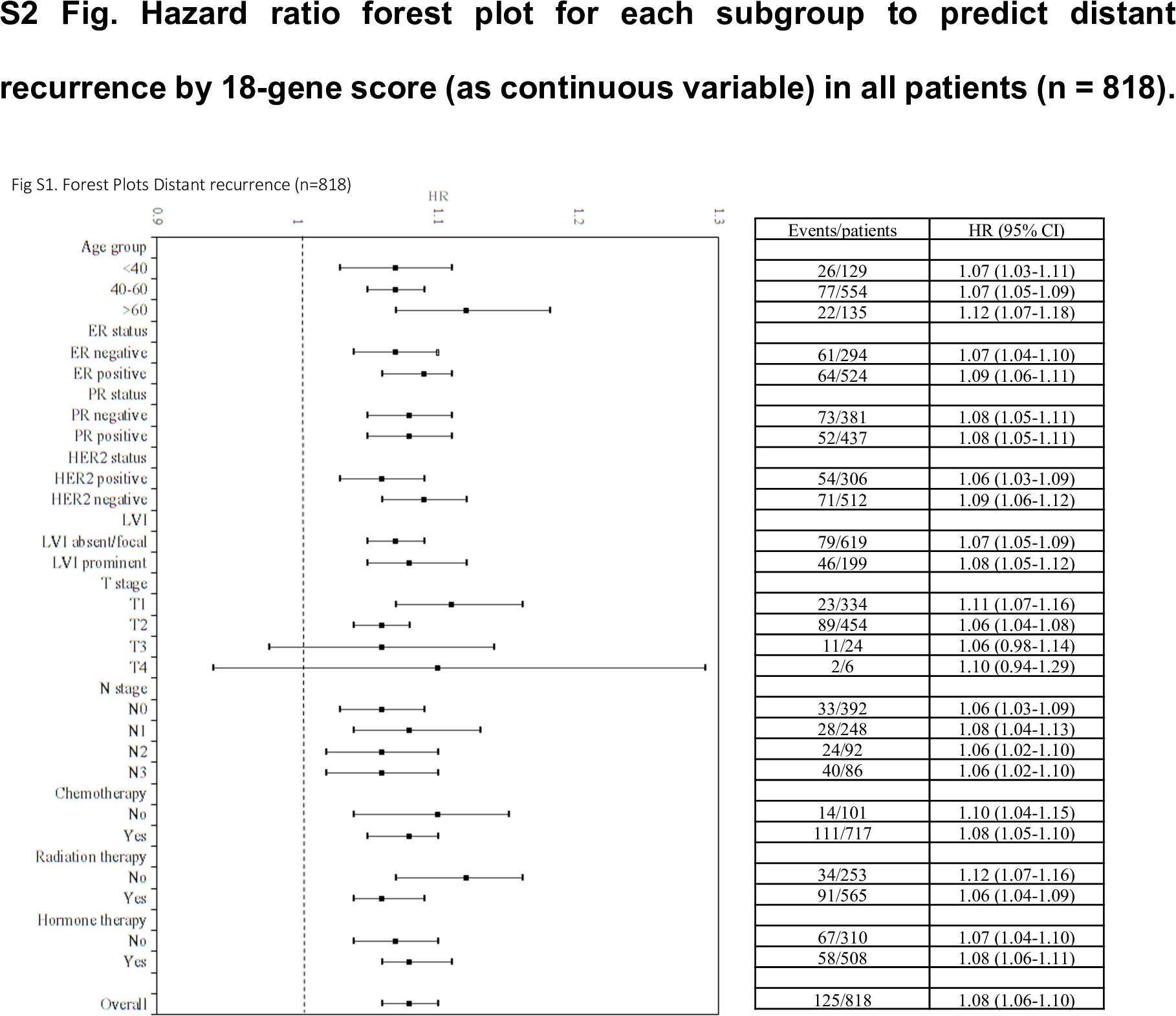

Supplement: S2 Fig — (TIF) [file pone.0184372.s005.tif]
